# Effect of health facility linkage with community using postnatal card on postnatal home visit coverage and newborn care practices in rural Ethiopia: A controlled quasi-experimental study design

**DOI:** 10.1371/journal.pone.0267686

**Published:** 2022-05-12

**Authors:** Yemane Berhane Tesfau, Tesfay Gebregzabher Gebrehiwot, Hagos Godefay, Alemayehu Bayray Kahsay

**Affiliations:** 1 College of Medicine and Health Sciences, Adigrat University, Adigrat, Ethiopia; 2 School of Public Health, College of Health Sciences, Mekelle University, Mekelle, Ethiopia; 3 Tigray Regional Health Bureau, Mekelle, Ethiopia; University of Oslo, NORWAY

## Abstract

**Background:**

Postnatal home visit has the potential to improve maternal and newborn health, but it remains as a missed opportunity in many low-and middle-income countries. This study examines the effect of health extension worker administered postnatal card combined with health facility strengthening intervention on postnatal home visit coverage, newborn care practices, and knowledge of newborn danger signs in rural Ethiopia.

**Methods:**

We employed quasi-experimental design using controlled before-and-after study in intervention and comparison districts of rural Tigray, northern Ethiopia. Training of health extension workers (HEWs) on postnatal home visit (PNHV), training of healthcare providers on maternal and newborn care, and capacity building of healthcare authorities on leadership, management and governance together with health system strengthening were the implemented interventions. Baseline (n = 705) and end line (n = 980) data were collected from mothers who delivered a year before the commencement of the actual data collection in the respective surveys. We used difference-in-differences (DiD) analysis to assess the effect of the intervention on PNHV coverage, essential newborn care practices and maternal knowledge of newborn danger signs.

**Results:**

A total of 1685 (100%) mothers participated in this study. In all districts, more than 1/3^rd^ of the mothers 633(37.57%) were in the age of 30–39 years. The difference-in-differences estimator showed an average of 23.5% increase in coverage of PNHVs within three days (DiD, p<0.001) and the provision of most postnatal contents significantly increased in the intervention district in the end line survey. The knowledge of at least three danger signs increased by 13.6% (p = 0.012).The DiD estimator showed an average of 27.6% increase to check the mothers for heavy bleeding (DiD, p = 0.011). This study also revealed that the checking of maternal blood pressure increased from 5.8% to 11.8% in the comparison districts and from 9.4% to 93.3% in the intervention district. The difference-in-differences estimator result showed a 9% difference in clean cord care practices among the participants (p = 0.025), 12.2% in skin to skin care (p = 0.022), and borderline significant increase in early initiation of breastfeeding (10.5%, p = 0.051).

**Conclusion:**

We conclude that the intervention package was effective in improving the coverage of PNHV, increase in knowledge of newborn danger sign and essential newborn care practices. Hence, further strengthening the linkages between health facilities and community is imperative to improve the coverage of essential lifesaving maternal and newborn care services by HEWs at home.

## Introduction

Improving maternal and newborn health service utilization at facility and community levels is one of the key challenges in low-income countries like Ethiopia. This issue is particularly critical in improving the coverage of postnatal care.

Globally, more than half a million reproductive age mothers die every year due to complications arising from pregnancy and childbirth, where almost all these deaths were in low-income countries [1]. Around 2/3^rd^ of maternal and newborn deaths occur in the early postpartum period in low- income settings where the sab-Saharan African countries take the lion-share of the majority of these deaths. Evidence showed that most maternal deaths (52.5%) occur in the postpartum period and most deaths under five (40.2–54.5%) occur in the neonatal period [2–4].

In Ethiopia, maternal and neonatal mortality remain the highest among the world, with 412/100,000 and 30/1,000, respectively. Though, trends of the Ethiopian demographic health surveys (EDHS) report has shown a continuous decline in infant and under-five child mortality, still, the trend among the neonatal mortality has remained stable since the 2016 EDHS [5]. In addition, it has been known that under-five mortality rate has declined significantly by two-third (67%) from 204/1000 live births in 1990 to 59/1000 live births in 2019. This reduction in child mortality was primarily after the first year of life indicating that the progress of Ethiopia in reducing neonatal death has been less successful [6].

Despite better achievements of antenatal care (ANC) and skilled delivery, however, postnatal care (PNC) utilization at health facilities still remains low due to enormous reasons such as poor attention, unavailability of the services, inaccessibility, poor quality of health services, socio-cultural beliefs, poor awareness of women on danger signs, lack of awareness about the availability of the service, and distance [7–19]. Studies also showed that significant proportions of mothers who prefer to return home or discharged within a few hours after delivery which makes them preclude the required care [20–22].

In countries with high new-born mortality, early postnatal home visits (PNHVs) by community health workers had brought a change in reducing neonatal and maternal deaths and improving newborn care practices such as timely initiation of breast feeding, clean cord care, thermal care and delayed bathing [23–28].

Based on the experiences and evidence from South Asian trials, in 2009, World Health Organization (WHO) and United Nations Children’s Fund (UNICEF) issued a joint statement recommending postnatal home visits (PNHVs) [29]. Among the 75 countries included in the Countdown to 2015 report, 59 have policies to deliver such home visits within one week of birth [30, 31]. High coverage of home visits by community health workers (CHWs) during pregnancy and postnatal period plays vital role in improving essential lifesaving practices and reducing newborn mortality [26]. However, their coverage remains low. Therefore, programs need to investigate the most appropriate and efficient ways to reach families and promote newborn care practices.

The health extension program of Ethiopia, which was launched in 2003, contributed intensive community mobilization towards the utilization of antenatal care and institutional delivery. In rural Ethiopia, HEWs were responsible to work at the health post and visit house to house on village based approach. They have been supervised by health workers who were assigned at health centers within the district. The HEWs are linked to their local community, to whom they serve and receive referrals. In Ethiopia, evidence show low coverage of PNHV by HEWs, inadequate knowledge of newborn danger signs and poor newborn care practices [22, 32, 33]. Particularly, the coverage of PNHV based on the recommended schedule is extremely low in Ethiopia.i.e. 6.6%, 0.85%, 0.71% within 24 hours, three days, and seven days respectively [22, 34].

Cognizant of this fact, the government of Ethiopia replenished its commitment by developing packages for optimizing newborn care practices and reduce newborn mortality from 33/1000 live births to 21/100 live births in 2024/2025. Moreover, the Sustainable Development Goal (SDG) puts an ambitious target of achieving MMR of 70 per 100,000 live births in 2030 [35]. Therefore, in order to track future targets improving PNHV coverage by HEWs, maternal knowledge on newborn danger signs and essential newborn care practices is vital. Hence, to improve those maternal and newborn health services, Mekelle University School of Public Health with support from Tigray regional health bureau and Tigray KMC project implemented an intervention from August 10, 2019– August 20, 2020. Thus, this is the first study that serves to evaluate the effect of the intervention on postnatal home visit coverage, knowledge on newborn danger signs and essential newborn care practices in rural Tigray, northern Ethiopia.

## Materials and methods

### Design

We employed a quasi-experimental study design using controlled before-and-after study in the intervention and comparison districts. The baseline survey was conducted in 2018 and the end line survey in 2020.

### Study setting

We conducted the study in four districts of Southeastern zone of Tigray regional state. The selections of these districts were based on overall representation in relation to child and neonatal mortality indicators, and progress in implementation of the integrated management of childhood illness programs. One district was selected out of the four districts for the implementation of the intervention in consultation with the regional health bureau and the district health offices. The selection of the district was purposive and determined based on maternal and newborn service indicators for the districts and district health office interest in participation. It was also convenient to select the district by the researchers because of the easy access from Mekelle, the capital city of the region. The four districts surround the capital city (Mekelle) of Tigray region.

The zone serves a population in excess of 567,700 inhabitants with the total households estimated at 129,031. With regard to the number of health professionals, there were 731 health care providers in the zone out of which 183 were HEWs [36].

At least one antenatal care coverage of the zone was, 97%, facility delivery was 89.2%. Out of all facility deliveries, only 18.2% of them had a minimum facility stay of 24 hours post-delivery. Postnatal home visit coverage by the HEWs within three days was 14.5% with only 0.71% mothers receiving the scheduled three postnatal home visits: within 24 hours, three days, and seven days [22].

### Sample size and sampling technique

Before the implementation of the intervention, community based survey was conducted to assess the coverage of postnatal home visits by the HEWs among 705 postnatal mothers in the proportionally selected 30 villages.

The sample size was determined using a two-sided Z test of the difference between proportions with 80% statistical power, a 5% significance level. The outcome of interest used in the calculation of the sample size was the proportion of mothers who received PNHV within three days which was 14.5% from the baseline survey in northern Ethiopia. By assuming the effect size to be increased by 10% after a 1year implementation of the intervention with un equal cluster size evaluation, the design effect was 1.81 which was calculated by considering the intra-class correlation (roh) = 0.05 and coefficient of variation (CV) = 0.12 [37–39]. With a non-response rate of 10%, 980 mothers were sampled for the end line survey. A total of 1685 mothers were included both for the intervention and comparison districts.


n=(Zα2+Zβ)2((P1(1−P1)+P2(1−P2)))(P1−P2)2

A multi-stage sampling technique was applied to select the study participants. In the first stage we selected 30 villages to realize the total sample size. All households’ of mothers having delivered in the 12 months preceding the baseline and end line were listed and registered by the HEWs in communication with Women development group (WDG) leaders in the selected villages. We sampled a total of 373 mothers in the intervention and 1312 mothers in the comparison districts by using simple random sampling techniques.

### Ethics approval and consent to participate

The study was approved by the Institutional Review Board of Mekelle University, College of Health Sciences (No.1437/2018). Verbal consent was obtained from the study participants after explaining the objectives of the study and the use of verbal consent was approved by the ethics committee. Privacy and confidentiality of the respondents were maintained during data collection and analysis. Mothers and newborns in the comparison group received the routine care services.

### Data collection procedure

We performed a multi-stage sampling strategy to select communities as sampling units in proportion to their population size. Households meeting the eligibility criteria were randomly selected for the interviews from each sampling unit. Baseline and end line data were collected in 2018 and 2020, respectively with an interviewer-administered structured questionnaire that was adapted from Ethiopia demography health survey (EDHS) and the last 10 km (L10K) survey [40, 41]. The tool contains items regarding socio-demographic, status towards model household, community based participations like pregnant women forum, participation in WDG, community health insurance membership, availability of HEW’s cell phone at home, time taken to visit the household, ANC attendance (both facility and home), place of delivery, birth notification, attendants at birth, postnatal visits, contents of PNC provided, maternal knowledge on postnatal danger signs and essential newborn care practices. It was initially prepared in English and then translated into the local language (Tigrigna) and translated back to English by language experts. The questionnaire was pre-tested prior to the commencement of actual data collection outside the study districts. A total of 20 field workers (BSc and above in nursing, and midwifery) were recruited for the data collection and 2 days training was given for the data collectors. Four field supervisors had also participated in the data collection. The primary outcome measures were: PNHV by HEWs and essential newborn care practices. The secondary outcome measures were postnatal care contents and maternal knowledge on newborn danger signs.

### Intervention

By considering the quantitative (low coverage of scheduled PNHV) [22] and qualitative finding (poor attention of healthcare authorities, lack of effective supervision, poor functional linkages, inadequate logistics and supplies, poor community participation and support), an intervention was designed by the authors in consultation with the regional health bureau and the district health offices. Hence, to improve those maternal and newborn health services, Mekelle University School of public health with support from Tigray regional health bureau and Tigray KMC project implemented an intervention from August 10, 2019– August 20, 2020.

Healthcare providers in the intervention district were trained on essential newborn care practice and postpartum care at facility and to link the mothers with and for the provision of PNHV by the HEWs using postnatal card. The postnatal card contains both maternal and newborn postnatal care contents documented in a separate section to be addressed by health extension workers (HEWs) on consequent visits that are recommended by the world health organization (WHO) (within 24 hours, 3^rd^ day, 7^th^ day and 42^nd^ days) (**S1 File**).

All responsible healthcare providers in the maternal, newborn and child health (MNCH) clinic were expected to counsel the mothers about the postnatal danger signs and inform them to have awareness about the presence of the service at home before the mother discharged from the facility and hence, linked them with HEWs. Evidences also demonstrated that there is a positive association between any recalling mechanisms for an appointment like SMS and healthcare utilization [11, 42, 43].

We provided training for all HEWs (39) to conduct home visits during pregnancy and postnatal period. In addition, the intervention included strengthening the health facilities, mainly through training of midwiferies, nurses and health officers who were assigned in the MNCH clinic about the provision of essential postnatal contents within 24 hours at facility and linkage with HEWs (a total of 36). Six days training was provided for the facility directors and supervisors, regional MNCH expert, district health office director, and MNCH expert about leadership, management and governance (15 participants) with emphasis to postnatal care services. It also included mobilization of necessary postnatal care supplies for all the health facilities in the intervention district.

Following training, HEWs were instructed to create a register of pregnant women in their catchment areas and update the list every month through home visits and discussions with WDG leaders to identify current pregnancies and mobilize the community to maximize the demand for postnatal care at home. WDG leaders were expected to conduct pregnant women forum and report to HEWs on monthly basis. HEWs were expected to make at least 3 postnatal home visits for mothers and newborns (on days 1, 3 and 7). Mothers were expected to deliver and stay at least 24 hours in health facility. After having received the essential postnatal contents for their newborn and themselves, they were supposed to discharge having postnatal card labeled with postnatal contents for the mother and newborns. For those mothers who delivered in facilities other than the intervention district and at home, they were expected to receive their 1^st^ PNHV from HEWs within 24 hours after delivery. The HEWs were instructed to conduct PNHV using postnatal card and were expected to provide a package of essential lifesaving contents for the mother and/newborns.

The implementation of the study was undertaken with the support of Tigray regional health bureau, Mekelle University, and the KMC project.

Supportive supervision was conducted based on the recommended schedule i.e. every month [44] by the supervisors from the respective health facilities in accordance with the existing district health service structure. We also planned quarterly meetings with HEWs, district management bodies and regional health bureau MNCH representatives. However, only a one day performance review and refresher training meeting were organized after 4 months of implementation. Implementation status was followed through the supportive supervision of the district supervisors and the research team. During the filed visit the supervisors observed and checked the provision of essential postnatal care both for the mother and newborns within 24 hours in health facilities. They also checked whether the postnatal card is filled and completed with the necessary contents that are supposed to deliver at health facilities within 24 hours. In the community, supervisors checked whether the postnatal card was filled with the recommended schedule. They also interviewed with some of the mothers about the provision of PNHV. As part of the monitoring system, all health posts were provided with uniform postnatal registration books which were developed by the research team. The outcome evaluation of the intervention was conducted through household surveys in the end line in between August and September 2020.

### Outcome measures

We used the WHO recommendation of four postnatal care visits at day 1, i.e., within 24 hours of birth, day 3 (second visit), between days 7–14 (third visit) and week 6 (fourth visit). The coverage of postnatal home visits was defined as the percentage of women and/or newborns that were visited at home within 3 days after delivery. We measured newborn care practices with five items (initiation of breast feeding within one hour, skin to skin contact care, clean cord care/applied nothing (harmful) to the cord, bathing the newborn ≥24 hours after birth, and provision of colostrum to the newborn).

Maternal knowledge on newborn danger signs was measured with 11 items. Participants were asked to mention spontaneously the key danger signs of newborns (Red eye/pus draining from the eye, cord bleeding/pus draining from the cord, jaundice, low body weight/preterm, low body temperature, fever, fast breathing, shortness of breathing, poor feeding, movement when stimulated/no movement even when stimulated, and convulsion). Those mothers who had mentioned more than or equal to three newborn danger signs were considered as having good knowledge about newborn danger signs [45].

### Statistical analysis

The data was entered in to SPSS version 23 and exported to Stata software (version 14.0) for the analyses. Descriptive statistics was performed by computing frequencies across the intervention and comparison districts. We employed difference-in-differences (DiD) analysis to assess the contribution of the intervention package towards PNHV coverage, newborn care practices, knowledge of postnatal danger signs and postnatal care contents provided. The DiD analysis is based on comparing the percentage differences in the intervention district (before and after the intervention) to differences in the comparison districts and assumes that trends in both groups are the same in the absence of the intervention. Pearson Chi square tests and t- tests were used to compare differences between intervention and comparison districts/ we conducted test of homogeneity in the districts from the baseline to the end line surveys. We created a model that included dummy variables for the treatment and time variables: 0 for the comparison district, 1 for the treatment district); and (0 for the base line, 1 for the end line surveys).

## Result

### Socio demographic characteristics of the respondents

A total of 1685 (100%) mothers participated in this study (705 mothers at baseline and 980 mothers at end line). More than one third of the mothers 633(37.57%) were in the age of 30–39 years. The mean age of the respondents in the intervention district was 28.34 ±5.72 years (27.71 years in the base line and 28.98 years at end line); and their mean age in the comparison district was 27.70 ±5.80 years (27.49 years in the base line and 27.91 years at the end line). Three-fifth, 1021 (60.6%) of the mothers had completed at least primary education and majority 1,454 (86.3%) of them were housewives. More than two-third, 1,168 (69.32%) of respondents lives less than 30 minutes walking distance to a nearby health facility (263 (70.5%) in the intervention and 905 (69%) in the comparison group). No significant difference between study districts in any survey rounds was found (**Table 1)**.

**Table 1:** Sociodemographic characteristics of participants at baseline and end line surveys in intervention and comparison areas.

Characteristics	Baseline household survey					End line household survey		
	Comparison n (%)		Intervention n (%)		p-value	Comparison n (%)	Intervention n (%)	p- value
**Read and write simple sentences**								
No	165(29.1)			38(27.5)		217(29.1)	76(32.34)	
Yes	402(70.9)			100(72.5)	0.716	528(70.9)	159(67.66)	0.348
**Educational status**								
No education	230(40.56)			54(39.13)		283(37.99)	97(41.28)	
Primary education	204(35.98)			46(33.33)	0.595	262(35.17)	83(35.32)	0.519
> = Secondary education	133(23.46)			38(27.54)		200(26.85)	55(23.40)	
Mother’s occupation								
House wife	504(88.89)			117(84.78)	0.427	633(84.97)	200(85.11)	0.742
**Mother’s age in years**								
18–24	198(34.92)			47(34.06)		249(33.42)	58(24.68)	
25–29	144(25.40)			39(28.26)		190(25.50)	67(28.51)	
30–39	205(36.16)			45(32.61)	0.692	282(37.85)	101(42.98)	0.095
40–49	20(3.53)			7(5.07)		24(3.22)	9(3.83)	
**Parity**								
Primipara	129(22.75)			35(25.36)	0.52	182(24.43)	43(18.30)	0.051
Multipara	438 (77.25)			103 (74.64)		563 (75.57)	192 (81.7)	
**Distance to nearest health facility**								
<30 minute		405(71.43)		96(69.57)		500(67.11)	167(71.06)	
30minute-<1 hour		130(22.93)		30(21.74)	0.411	189(25.37)	56(23.83)	0.354
> = 1 hour		32(5.64)		12(8.70)		56(7.52)	12(5.11)	

### Maternal and newborn health related characteristics of respondents

In general, 684 (97%) and 964 (98%) of the participants had received at least one ANC visit at health facilities in the baseline and end line surveys, respectively. More than one-third, 242 (34.3%) and about two-third, 657(67%) of the mothers received ≥4 ANC visit at health facilities in the base line and end line surveys, respectively. Overall, 790 (46.9%) of the respondents were members of community based health insurance (CBHI) with 229 (32.5%) and 561 (57.2%) participants in the base line and end line surveys, respectively. Significant differences were observed between intervention and comparison sites for membership of community based health insurance (CBHI) at baseline and participation of mothers in the pregnant women forum, and timing of home visit by HEWs during pregnancy in the end line (**Table 2)**.

**Table 2:** Maternal and newborn health related characteristics of respondents at baseline and end line surveys in intervention and comparison areas.

Characteristics	Baseline survey					End line survey		
	Comparison		Intervention		P- value	Comparison	Intervention	P- value
	n (%)		n (%)			n (%)	n (%)	
**ANC at facility**								
Yes	549 (96.8)		135(97.8)		0.535	732(98.3)	232(98.3)	0.621
No	18 (3.2)		3(2.2)			13(1.7)	3(1.3)	
**Gestational age at 1**^**st**^ **ANC contact at facility**								
≤12 weeks	144 (26.2)		33 (24.5)			200 (27.5)	62 (26.7)	
13–27 weeks	393 (71.6)		101 (74.8)		0.477	517 (71.0)	167 (72.0)	0.943
≥28weeks	12 (2.2)		1 (0.7)			11(1.5)	3 (1.3)	
**Place of delivery**								
Facility	501 (88.4)		126(91.3)		0.323	683(91.7)	218(92.8)	0.593
Home	66 (11.6)		12(8.7)			62(8.3)	17(7.2)	
**PNC visit at facility**								
No PNC visit		562 (99.1)	97 (70.3)			690 (92.6)	214 (91.1)	
Within one day		1(0.2)	0		0.874	5 (0.7)	2 (0.8)	0.688
Within 2–3 days		1(0.2)	0			14 (1.9)	7 (3.0)	
Within 4–7 days		3 (0.5)	0			33 (4.4)	12 (5.1)	
**Member of CBHI**								
Yes		201 (35.4)	28 (20.3)	0.001		424 (56.9)	137(58.3)	0.708
No		366 (64.6)	110 (79.7)			321 (43.1)	98 (41.7)	
**Participation in Pregnant women forum**								
Yes		310 (54.7)	74 (53.6)	0.824		385 (51.7)	141 (60.0)	0.026
No		257 (45.3)	64 (46.4)			360 (48.3)	94 (40)	
**ANC visit at home by HEWs**								
No visit		240 (42.3)	47 (34.1)			332 (44.6)	35 (14.9)	
1^st^ trimester		38 (6.7)	9 (6.5)	0.22		66 (8.9)	20 (8.5)	<0.001
2^nd^ trimester		249 (43.9)	74 (53.6)			339 (45.5)	174 (74.0)	
3^rd^ trimester		40 (7.1)	8 (5.8)			8 (1.1)	6 (2.6)	

### Effect of the intervention on maternal and newborn PNHV coverage

The difference-in-differences estimator shows an average 23.5% increase in coverage of PNHVs within three days in the intervention district (DiD, p<0.001). Overall, More than one-third 574 (34.1%) of the respondents received postnatal home visit within the postnatal period with 170 (24.1%) and 404 (41.2%) in the baseline and end line surveys, respectively (**Table 3**).

**Table 3:** Effect of the intervention on maternal and newborn PNHV coverage.

Indicator	Base line			End line				P- value
	Comparison n (%)	Intervention n (%)	Diff	Comparison n (%)	Intervention n(%)	Diff	DiD	
PNHV within three days	88 (15.5)	14 (10.1)	-5.4	147 (19.7)	89 (37.9)	18.1	23.5	<0.001
PNHV within seven days	115 (20.3)	21 (15.2)	-5.1	217 (29.1)	122 (51.9)	22.8	27.9	<0.001
PNHV within 42 days	138 (24.3)	32 (23.2)	-1.2	255 (34.2)	151 (64.3)	30.0	31.2	<0.001

Diff = Single difference.

### Effect of the intervention on provision of maternal postnatal contents at home

Overall, the provision of maternal postnatal contents significantly increased in the intervention district in the end line survey (**Table 4**).The DiD estimator shows an average 27.6% increase in checking of the mothers for heavy bleeding in the intervention district (DiD, p = 0.011). The study also revealed that checking of maternal blood pressure (BP) increased from 5.8% to 11.8% in the comparison districts and from 9.4% to 93.3% in the intervention district, indicating a statistically significant contribution of the intervention by 77.9% (DiD p < 0.001).

**Table 4:** Effect of the intervention on provision of maternal postnatal contents at home.

Variable		Base line						End line							p-value
		Comparison n (%)		Intervention n (%)		Diff		Comparison n (%)		Intervention n (%)		Diff		DiD	
Maternal contents															
Underwent body examination	31 (22.5)		6 (18.8)		-3.7		61 (23.9)		47 (31.5)		7.6		11.3		0.24
Checked heavy bleeding	57 (41.3)		10 (31.2)		-10.1		87 (34.1)		77 (51.7)		17.6		27.6		0.011
Measured their body Temperature	15 (10.9)		4 (12.5)		1.6		22 (8.6)		92 (61.7)		53.1		51.5		<0.001
Measured their blood pressure	8 (5.8)		3 (9.4)		3.6		30 (11.8)		139 (93.3)		81.5		77.9		<0.001
Counseled about personal hygiene	38 (27.5)		10 (31.2)		3.7		90 (35.3)		99 (66.4)		31.1		27.4		0.009
Counseled about family planning	28 (20.3)		6 (18.7)		-1.5		90 (35.3)		105 (70.5)		35.2		36.7		<0.001
Checked TT immunization	6 (4.3)		0.0		-4.3		47 (18.4)		35 (25.5)		7.1		11.4		0.149
Counseled breastfeeding	45 (32.6)		9 (28.1)		-4.5		98 (38.4)		110 (73.8)		35.4		39.9		<0.001
Counseled about own feeding	47 (34.1)		10 (31.2)		-2.8		97 (38.0)		120 (80.5)		42.5		45.3		<0.001
Checked for Iron intake	4 (2.9)		0		-2.9		73 (28.6)		44 (29.5)		0.9		3.8		0.66

Diff = Single difference.

### Effect of the intervention on provision of newborn postnatal contents at home

**Fig 1:**
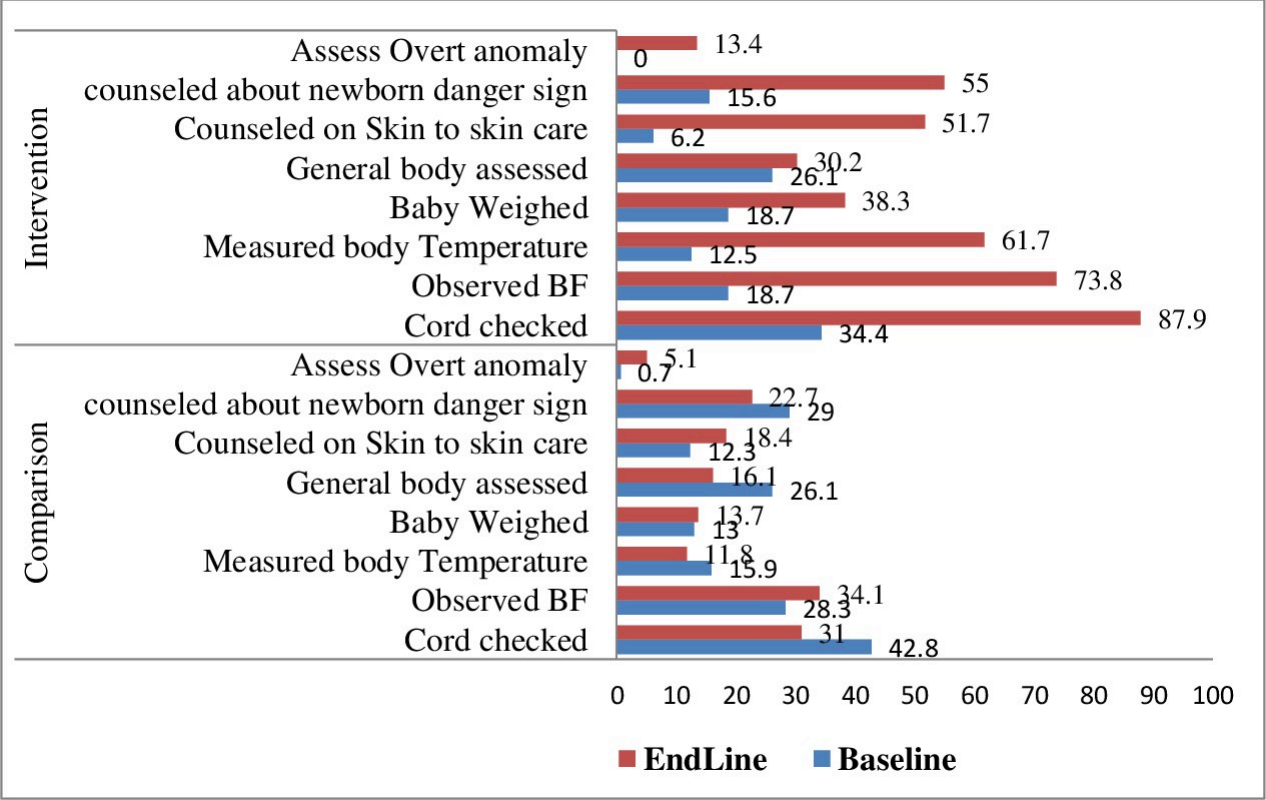
Proportion of newborn postnatal contents provided by HEWs during PNHV.

The difference-in-differences estimator revealed that the provision of the essential lifesaving newborn contents at home were improved (**Fig 1**).

### Effect of the intervention on newborn danger signs

Overall, three of ten, 506 (30.03%) of the participants mentioned at least three newborn danger signs (95% CI: 27.84–32.22). The knowledge of at least three danger signs increased by 13.6% (p = 0.012). More than 4/5^th^, 1400 (83.1%) of the participants were able to recall at least one newborn danger sign (494 (70.1%) and 906 (92.4%) in the base line and end line surveys, respectively. The most commonly mentioned newborn danger sign was fever, 948 (56.3%) followed by poor feeding of the newborn, 725 (43.03%). The DiD estimator shows an average increase in knowledge of cord bleeding/ presence of pus, low body weight/and pre-term, low body temperature, and fever with 15% (DiD, p = 0.004), 16% (DiD, p<0.001), 11.4% (DiD, p = 0.003), and 13.3% (DiD, p = 0.023), respectively in the intervention district (**Table 5**).

**Table 5:** Effect of the intervention on knowledge of neonatal danger signs.

Variables	Baseline			End line			DiD	p-value
	Comparison n (%)	Intervention n (%)	Diff	Comparison n (%)	Intervention n (%)	Diff		
Red eye/pus draining	42 (7.4)	7 (5.1)	-2.3	78 (10.5)	22 (9.4)	-1.1	1.2	0.721
Cord bleeding/pus on the cord	125 (22.0)	27 (19.6)	-2.5	176 (23.6)	85 (36.2)	12.5	15	0.004
Jaundice	18 (3.2)	4 (2.9)	-0.3	100 (13.4)	28 (11.9)	-1.5	-1.2	0.717
Low body weight/preterm	6 (1.1)	0	-1.1	70 (9.4)	57 (24.3)	14.9	15.9	<0.001
Low body temperature	37 (6.5)	6 (4.3)	-2.2	96 (12.9)	52 (22.1)	9.2	11.4	0.003
Fast breathing	44 (7.8)	11 (8.0)	0.2	143 (19.2)	39 (16.6)	-2.6	-2.8	0.499
Shortness of breathing	51 (9.0)	12 (8.7)	-0.3	166 (22.3)	54 (23.0)	0.7	1.0	0.823
Fever	244 (43.0)	61 (44.2)	1.2	463 (62.2)	180 (76.6)	14.4	13.3	0.023
Poor feeding	216 (38.1)	52 (37.7)	-0.4	348 (46.7)	109 (46.4)	-0.3	0.1	0.989
Movement/no movement	21 (3.7)	8 (5.8)	2.1	75 (10.1)	24 (10.2)	0.1	-1.9	0.541
Convulsion	59 (10.4)	7 (5.1)	-5.3	84 (11.3)	25 (10.6)	-0.7	4.6	0.746

### Effect of the intervention on newborn care practices

Respondents reported an increase in the essential newborn care practices from the baseline survey including initiation of breast feeding within one hour of birth (3.5% vs.13.9%), colostrum given to the newborn (0.6% vs.1.6%), skin to skin care (3.5% vs. 15.7.%), frequency of breast feeding (5.7% vs. 13.3%), and delayed bathing to 24 hours (0.6% vs. 6.2%). The DiD estimator shows a 9% difference in clean cord care practices (p = 0.025). The DiD analysis of the newborn care practices shows a statistical significant increase in skin to skin care (12.2%, p = 0.022), and borderline significant increase in early initiation of breastfeeding (10.5%, p = 0.051) **(Table 6)**.

**Table 6:** Effect of the intervention on newborn care practices.

Newborn care practices	Baseline		End line		Pre Diff	Post Diff	DiD	p-value
	Comparison (%)	Intervention (%)	Comparison (%)	Intervention (%)				
Initiated BF within an hour	309 (54.5)	80 (58.0)	565 (75.8)	211 (89.8)	3.5	13.9	10.5	0.051
Colostrum given to baby	525 (92.6)	129 (93.5)	698 (93.7)	224 (95.3)	0.9	1.6	0.7	0.803
Frequency of BF ≥8 times	395 (69.7)	104 (75.4)	560 (75.2)	208 (88.5)	5.7	13.3	7.6	0.14
Skin to skin care	420 (74.1)	107 (77.5)	511 (68.6)	198 (84.3)	3.5	15.7	12.2	0.022
Delayed bathing	510 (89.9)	125 (90.6)	683 (91.7)	230 (97.9)	0.6	6.2	5.6	0.091
Non recommended cord care	79 (13.9)	17 (12.3)	111 (14.9)	10 (4.3)	-1.6	-10.6	-9.0	0.025

## Discussion

To the best of our knowledge, this is the first implementation study that has evaluated the effect of health facility linkage with community using postnatal card and reported postnatal home visit coverage and newborn care practices in rural districts of northern Ethiopia.

The results of this study showed that the intervention was successful in improving the coverage of PNHVs. Indeed, a significant improvement was observed across all the provision of postnatal contents of the mother and newborns. Moreover, the intervention package improved knowledge of newborn danger signs and practices of essential newborn care among the mothers.

Previous studies demonstrated that increasing demand for and access to ANC and skilled delivery were the major challenges in improving maternal and newborn care services in Ethiopia [46]. Our findings, however, suggests that improving PNHV coverage and newborn care practices is possible with this intervention as there were high coverage of ANC and skilled delivery which eases for the continuum of care.

The difference-in-differences estimator shows an average of 23.5% increase in coverage of PNHVs within three days in the intervention district. Although this study was conducted in one district, the finding suggests that in rural and low -income community like Ethiopia, where facility postnatal visit is almost non-existent, HEWs play a significant role in improving maternal and newborn health, especially the missed service i.e. postnatal care if interventions are managed appropriately. Similar improvements in coverage of postnatal care have been observed in other parties of rural community, where the coverage within 48 hours was improved [25, 47–49]. Improving maternal knowledge on newborn danger signs is important for seeking maternal and newborn health services and increase demand for HEWs at home, which in turn improves essential live saving newborn care practices.

The intervention packages had significant positive effects on knowledge of newborn danger signs, but there were also improvements in the comparison districts during the implementation period. The proportion of mothers who mentioned at least three newborn danger signs increased by 13.6%, according to the DiD estimator. Similarly, findings from other community based studies showed improvements in knowledge of newborn danger signs [50]. Knowledge on cord bleeding/ presence of pus on the cord, low body weight and preterm, low body temperature, and fever among the respondents showed statically significant improvements.

Studies showed that neonatal and maternal morbidity and mortality can be significantly reduced with basic and low cost interventions if managed appropriately and with high coverage of PNHV [51–53].

Evidence showed that home-based counseling strategy using community volunteers can improve newborn care behaviors in rural communities [48]. In this study, the intervention had improved for all the essential newborn care practices. However, the DiD analysis showed a statistical significant increase in skin to skin care, decreased non recommended cord care, borderline significant increase in early initiation of breastfeeding and delayed bathing. Similar improvements in newborn care practices also observed in Sub-Saharan Africa and Asia where home visits by community health workers during pregnancy and the early postpartum period Moreover, engagement with WDG contributed to improvements in essential newborn care practices [25, 54–58]. Despite improvements observed in the essential newborn care practices, the absolute percentage changes were minimal due to probably the ingrained cultural practices and timing of counseling the mothers in the rural community. Similar findings were reported from maternal and newborn health related interventions [37, 48, 51, 59–61].

### Study limitations and strengths

This community-based experiment helps to overcome the external validity limitations of “facility-based” study by enrolling random samples of well-defined population. Other threats to external validity, however, may persist even if a representative sample of a well-defined population participates in this study. The use of a quasi-experimental design may not also control confounders that are often best handled through randomization. In conducting controlled before-and-after study, it is necessary to select the comparison district for conducting the DiD analysis, which is as similar as the intervention district before the intervention is implemented using different matching techniques. However, in this study we used chi-square test and t-tests to compare observable differences between intervention and comparison districts at baseline and end line surveys. There could be also recall bias as data were collected from mothers having a live birth preceding a year before each survey. There is also the possibility of social desirability bias among the mothers, especially when reporting newborn care practices. The DiD analysis may have limited external validity for the participants that have significant difference in their characteristics. Our finding might also be influenced by the relatively short intervention period especially for some newborn care practices.

## Conclusions

This study indicated that the intervention package was effective in improving the coverage of PNHV, increase in knowledge of newborn danger sign and essential newborn care practices. Hence, further strengthening the linkages between health facilities and community is imperative to improve the coverage of essential lifesaving maternal and newborn care services by HEWs at home. Therefore, policy makers and healthcare authorities should give emphasis to health facility-community linkage and community awareness about the presence of the service at home so that HEWs are notified early for all births in a way that could enhance PNHV demand. Assisting in capacity building and functioning of healthcare providers, healthcare authorities and community health workers to improve PNHV through training and the availability of uniform guidelines is crucial. Healthcare providers in health facilities should not miss the opportunity to provide pre-discharge PNC that could reduce the need for very early PNHVs.

## Supporting information

S1 Dataset Baseline and end line dataset. (SAV)

S1 File PNC service card. Postnatal care service card.
(DOCX)

S2 File English version questionnaire. Baseline and end line survey instrument.
(DOCX)

## List of abbreviations

ANC: Antenatal Care
CHW: Community Health Worker
CV: Coefficient of Variance
DiD: Difference-in-Differences
EDHS: Ethiopia Demographic Health Survey
HEP: Health Extension Package
HEW: Health Extension Worker
KMC: Kangaroo Mother Care
L10K: Last Ten Kilometers
MMR: Maternal Mortality Ratio
MNCH: Maternal, Newborn and Child Health
NDSs: Newborn Danger Signs
PNC: Postnatal Care
PNHV: Postnatal Home Visit
SDGs: Sustainable Development Goals
TT: Tetanus Toxoid
UNICEF: United Nations Children’s Fund
WDG: Women’s Development Group
WHO: World Health Organization
