# Supplementary material for: Effect of health facility linkage with community using postnatal card on postnatal home visit coverage and newborn care practices in rural Ethiopia: A controlled quasi-experimental study design
Source: PLoS One. 2022 May 12;17(5):e0267686. doi: 10.1371/journal.pone.0267686 (PMC9098030; doi:10.1371/journal.pone.0267686)
Supplement: S1 File — Postnatal care service card. (DOCX) [file pone.0267686.s002.docx]

**POSTNATAL CARD**

| **Home based PNC services card** | | | | | | |
| --- | --- | --- | --- | --- | --- | --- |
| **Maternal section** | | | | | **Cell phone** | **__________** |
| **Name** | | **___________** | **Tabia** | **____________** | **Village/ sub-district** | **_________** |
| **Age** | | **___________** | **Date of delivery** | **_____________** | **Place of delivery** | **__________** |
| **Activity** | | **1^st^ day** | **3^rd^ day** | **7^th^ day** | **42^nd^ day** | **Remark** |
| Vaginal bleeding | | No/Yes | No/Yes |  |  |  |
| Temperature | | ______^0^C | ______^0^C | ______^0^C |  |  |
| Blood pressure | | _____mmHg | _____mmHg | _____mmHg |  |  |
| Abdominal pain | | No/Yes | No/Yes | No/Yes |  |  |
| Urine incontinence | | No/Yes | No/Yes | No/Yes | No/Yes |  |
| Counseling Family planning | | No/Yes | No/Yes | No/Yes | No/Yes |  |
| Counseling on EBF | | No/Yes | No/Yes | No/Yes | No/Yes |  |
| Assess Emotional wellbeing | | No/Yes | No/Yes | No/Yes | No/Yes |  |
| TT vaccine | | No/Yes | No/Yes | No/Yes | No/Yes |  |
|  | |  | **Newborn section** |  |  |  |
| Name of Newborn | _____________ | | Sex | _____________ | Age | ________ |
| Father's full name | _____________ | | Cell phone | _____________ |  |  |
| **Activity** | **1^st^ day** | | **3^rd^ day** | **7^th^ day** | **42^nd^ day** | **Remark** |
| Respiration rate | ________/minute | | ________/minute | ________/minute | ________/minute |  |
| Temperature | ______^0^C | | ______^0^C | ______^0^C | ______^0^C |  |
| Unable to suck | No/Yes | | No/Yes | No/Yes | No/Yes |  |
| Vomiting every thing | No/Yes | | No/Yes | No/Yes | No/Yes |  |
| No movement | No/Yes | | No/Yes | No/Yes | No/Yes |  |
| Lethargic or unconscious | No/Yes | | No/Yes | No/Yes | No/Yes |  |
| Convulsion | No/Yes | | No/Yes | No/Yes | No/Yes |  |
| Jaundice | No/Yes | |  |  | No/Yes |  |
| Clean cord care/ Chlorhexidane | No/Yes | | No/Yes | No/Yes | No/Yes |  |
| Weight | _________grams | | _______grams |  |  |  |
| Congenital anomalies | No/Yes | | No/Yes | No/Yes | No/Yes |  |
| BF initiation within an hour | No/Yes | |  |  |  |  |
| Bathing deferred to 24hs | No/Yes | |  |  |  |  |
| Immunization | BCG/Polio zero | |  |  | No/Yes |  |
|  |  | |  |  |  |  |
| HEW's cell phone:_ |  | |  |  |  |  |
