# Supplementary material for: Effect of health facility linkage with community using postnatal card on postnatal home visit coverage and newborn care practices in rural Ethiopia: A controlled quasi-experimental study design
Source: PLoS One. 2022 May 12;17(5):e0267686. doi: 10.1371/journal.pone.0267686 (PMC9098030; doi:10.1371/journal.pone.0267686)
Supplement: S2 File — Baseline and end line survey instrument. (DOCX) [file pone.0267686.s003.docx]

## Annex I: Participant information sheet and consent form (Quantitative, maternal part)

Participant information sheet**:**

Hello ----

My name is -----------. I am working temporarily as a data collector with Mekelle University College of health sciences school of PH, in a study “Essential lifesaving home based postnatal care services in rural Tigray, northern Ethiopia”.

**Duration of the interview**: the interview will be conducted in private and will take up to 30 minutes.

**Objective:** The objective of the present study is to **examine the effect of health extension worker administered postnatal card combined with health facility strengthening intervention on postnatal home visit coverage, newborn care practices, and knowledge of newborn danger signs** in rural Ethiopia.

And the overall purpose of the study is to improve the health of mothers and their newborns in the community. During the interview you will be asked some short questions about your socio-demographic back ground, reproductive and obstetric factors, your knowledge about postnatal care services, and current practice of PNC at home, knowledge of danger signs during postnatal period for your selves and the newborn.

**Confidentiality**: Your answers will be recorded on a survey questionnaire. No personal identifiers will be attached/ recorded to the interview. All the data obtained will be kept strictly confidential.

**Autonomy**: Your participation in the study is upon purely voluntary basis. During the interview period, if you fill inconvenient, you have full right to refuse to take part or interrupt the interview at any time. Your honest and genuine participation in responding to the questions prepared is very important and highly appreciated.

**Risks and benefits**: your participation in this study does not poses any harm or risk to your body/newborn’s body and you will not receive any direct benefit from this study, however,
you will help to increase the health of the mother and newborns by assessing the information particularly postnatal care services. What we learn from this study will be used to generate information necessary for the planning to improve, redesign and scale up the PNC programs in our country.

Consent form:

Would you be willing to participate? No _______ thank and stop here.

Yes _______proceed to the interview.

Interviewer's signature ________________________ Date __________________________

| Questionnaire Code | ___________ |
| --- | --- |
| Area identification | Woreda:________________ Kebelle:__________ |
| Personal | Interviewer name and signature:  Supervisor name and signature: |
| Date of visit | ______/_____/_____  DD/ MM/ YYYY |

### Part I: Socio demographic characteristics

| **No** | **Variables** | **Responses** | **Skip** |
| --- | --- | --- | --- |
| 101 | Current maternal age | _______years |  |
| 102 | How old were you when you first gave a live birth? | _______years |  |
| 103 | Are you able to read or write a simple sentence? | 1. No 2. Yes |  |
| 104 | What is the highest level of schooling you have ever attended? | 1. No education 2. Primary 3. Secondary 4. More than secondary |  |
| 105 | What is your occupation? | 1. House wife 2. Daily worker/laborer 3. Government employee 4. Private employee   99. Others specify |  |
| 106 | What is your religion? | 1. Orthodox 2. Muslim 3. Catholic 4. Protestant |  |
| 107 | How old were you when you first married? | _______years |  |
| 108 | How many times pregnant were you? (including those that did not end with a live births) | Enter the number |  |
| 109 | How many times have you given birth? (live birth) | Enter the number |  |
| 110 | Sex of Child | 1. Boy 2. Girl |  |
| 111 | What is the age of your youngest child? | Enter the number |  |
| 112 | What is the birth date of your child? | ______/_____/_____  DD/ MM/ YYYY |  |
| 113 | Total number of Children ever born? | Boys____ Girls ____Total___ |  |
| 114 | **Your position based on economic classification** | 1. Rich 2. Medium 3. Lower/poor 4. I do not want to talk |  |

### Part II. Awareness of and access to health services in the community

| 201 | In average, How long does it take you to go to health facility? | _____ Minutes | **Skip** |
| --- | --- | --- | --- |
| 202 | Have you heard of or do you know about voluntary community health worker (such as WDG) in your community? | 1. No 2. Yes | If no, Q206 |
| 203 | Are you a member of WDG? | 1. No 2. Yes | If no, Q205 |
| 204 | Are you a leader of WDG? | 1. No 2. Yes |  |
| 205 | In the previous 6 month did the WDG visit your home? | 1. No 2. Yes |  |
| 206 | Do you know about model households | 1. No 2. Yes | If no, Q209 |
| 207 | Have you graduated to model family? | 1=Yes (certified)  2=Yes (not yet certified)  3= No (on process)  4= Not at all |  |
| 208 | Do you want to become model family? | 1. No 2. Yes |  |
| 209 | Have you participated in the pregnant women forum | 1. No 2. Yes |  |
| 210 | Do you have WDG leaders or HEWs, cell phone? | 1. No 2. Yes |  |
| 211 | Are you member of community based health insurance (CBHI)? | 1. No 2. Yes |  |
| 212 | Have you heard of or do you know about the health extension worker? | 1. No 2. Yes | If no, Q217 |
| 213 | Have you visited by HEW during your last pregnancy? | 1. No 2. Yes | If no, Q217 |
| 214 | How many times? | Enter number |  |
| 215 | How old was your pregnancy at the first home visit by HEW? | Enter number |  |
| 216 | Have you received any information about postnatal cervices from HEW during pregnancy? | 1. No 2. Yes |  |
| 217 | ANC visit at health facility | 1. No 2. Yes | If no, Q221 |
| 218 | How many times | 1=1  2=2  3=3  4>=4 |  |
| 219 | What was the age of the pregnancy during first ANC visit at facility? | ------weeks |  |
| 220 | Have you received any information about postnatal cervices from health care provider during pregnancy? | 1. No 2. Yes |  |
| 221 | Birth order | 1=1 2=2 3=3 4=4  5=5 6=6 7=>7 |  |
| 222 | Place of delivery | 1=Hospital  2=Health center  3=Health post  4=Home/on the way | If at home, Q301 |
| 223 | For how long do you wait in the health facility after delivery? | 1. <6 hours 2. 6-11 hours 3. 12-23 hours 4. >=24 hours |  |

### Part II: Maternal and newborn postnatal care services

| 301 | Does the mother visit Health facility for PNC? | 1=Yes  0=No | If no, Q303 | | | | | | | | |
| --- | --- | --- | --- | --- | --- | --- | --- | --- | --- | --- | --- |
| 302 | Time of Postnatal visit within Postnatal period | _____Days after delivery |  | | | | | | | | |
| 303 | Did the health care provider assess the mother after delivery? | 1=Yes  0=No | If no, Q308 | | | | | | | | |
| 304 | If yes, at what time did you first check take place? | 1= <4 hours  2= 4-24 hours  3= 25 hour-3 days  4=73 hours-7 days  5= after 7 days  6= I do not know | If 3, 4, 5, 6  Q306 | | | | | | | | |
| 305 | Postnatal contents delivered for the mother within 24 hours |  | Mentioned | | | | Not mentioned | | | | |
|  |  | Assessed vaginal bleeding | 1 | | | | 0 | | | | |
|  |  | body temperature measured | 1 | | | | 0 | | | | |
|  |  | Pulse rate measured | 1 | | | | 0 | | | | |
|  |  | Blood pressure measured | 1 | | | | 0 | | | | |
|  |  | Conducted breast assessment | 1 | | | | 0 | | | | |
| 306 | Where did the first check take place? | 1. Home 2. Health post 3. Health center 4. Hospital |  | | | | | | | | |
| 307 | Type of health provider for newborn’s first  postnatal check-up | 1. Doctor 2. Nurse/midwifery/health officer 3. Health extension worker   99. Others |  | | | | | | | | |
| 308 | After the baby was born, did a **health worker** check on your baby? | 1. Yes 2. No |  | | | | | | | | |
| 309 | If yes, at what time did your baby’s first check take place? | 1= <4 hours  2= 4-24 hours  3= 25 hour-3 days  4=73 hours-7 days  5= after 7 days  6= I do not know | If 3,4,5,6, Q311 | | | | | | | | |
| 310 | Newborn care within 24 hours |  | Mentioned | | | | | Not mentioned | | | |
|  |  | Assessed Breast Feeding | 1 | | | | | 0 | | | |
|  |  | Assessed baby movement | 1 | | | | | 0 | | | |
|  |  | Assessed overt birth defect | 1 | | | | | 0 | | | |
|  |  | Conducted clean cord care | 1 | | | | | 0 | | | |
|  |  | Counseled about colostrum feeding | 1 | | | | | 0 | | | |
|  |  | Counseled about skin to skin care | 1 | | | | | 0 | | | |
|  |  | Conducted immunization | 1 | | | | | 0 | | | |
|  |  | Conducted eye care with TTC | 1 | | | | | 0 | | | |
|  |  | Measured birth weight | 1 | | | | | 0 | | | |
|  |  | Counseled bathing to defer 24.hrs | 1 | | | | | 0 | | | |
| 311 | Type of health provider for newborn’s first  postnatal check-up | 1. Doctor 2. Nurse/midwifery/health officer 3. Health extension worker   99. Others |  | | | | | | | | |
| 312 | Where did the first check take place? | 1. Home 2. Health post 3. Health center 4. Hospital |  | | | | | | | | |
| 313 | Did the WDG conduct PNHV within 24 hours | 1. No 2. Yes 3. I do not remembered |  | | | | | | | | |
| 314 | Did the HEW conducted PNHV? | 1. No 2. Yes | If no, Q326 | | | | | | | | |
| 315 | Time of first PNHV by HEW | 1. <4 hors 2. 4-24 hours 3. 25 hours- 3 days 4. 73 hours-7 days 5. After 7 days 6. I do not know |  | | | | | | | | |
| 316 | Did the HEW visited for the second time? | 1. No 2. Yes 3. I do not remembered | If no/ I do not remember, Q322 | | | | | | | | |
| 317 | Time of second PNHV by HEW | ____hours after delivery  ____days after delivery |  | | | | | | | | |
| 318 | Did the HEW visited for the third time? | 1. No 2. Yes 3. I do not remembered | If no/ I do not remember, Q322 | | | | | | | | |
| 319 | Time of third PNHV by HEW | ____hours after delivery  ____days after delivery |  | | | | | | | | |
| 320 | Did the HEW visited for the fourth time? | 1. No 2. Yes 3. I do not remembered | If no/ I do not remember, Q322 | | | | | | | | |
| 321 | Time of fourth PNHV by HEW | ____days after delivery |  | | | | | | | | |
| 322 | Who did the HEW talk to her she visit you at home? | 1. By her self 2. WDG leader 3. Mother 4. Husband 5. Others |  | | | | | | | | |
| 323 | What did the HEW do during PNHV to check on your health? **Do not read responses** | **(M = mentioned, NM= not mentioned)** | **M** | | | | | | **NM** | | |
|  |  | 1. Examined body | 1 | | | | | | 0 | | |
|  |  | 1. Checked breast | 1 | | | | | | 0 | | |
|  |  | 1. Checked for heavy bleeding | 1 | | | | | | 0 | | |
|  |  | 1. Counseled on danger signs | 1 | | | | | | 0 | | |
|  |  | 1. Counseled on breast feeding | 1 | | | | | | 0 | | |
|  |  | 1. Counseled on family planning | 1 | | | | | | 0 | | |
|  |  | 1. Counseled on nutrition | 1 | | | | | | 0 | | |
|  |  | 1. Counseled about personal hygiene | 1 | | | | | | 0 | | |
|  |  | 1. TT immunization checked | 1 | | | | | | 0 | | |
|  |  | 1. check Iron intake | 1 | | | | | | 0 | | |
|  |  | 1. Measured B/P | 1 | | | | | | 0 | | |
|  |  | 1. measured Temperature | 1 | | | | | | 0 | | |
|  |  | 1. Counseled on safer sex including use of Condoms |  | | | | | |  | | |
|  |  | 99. Others | | | | | | | | | |
| 324 | What did the HEW do during that visit to check on the health of baby? **Do not read responses. Multiple responses are possible.** | **(M = mentioned, NM= not mentioned)** | **M** | | | | | | **NM** | | |
|  |  | 1. Generally examined/looked at baby’s body | 1 | | | | | | 0 | | |
|  |  | 1. Weighted baby | 1 | | | | | | 0 | | |
|  |  | 1. Checked cord | 1 | | | | | | 0 | | |
|  |  | 1. Counseled on breastfeeding | 1 | | | | | | 0 | | |
|  |  | 1. Observed breastfeeding | 1 | | | | | | 0 | | |
|  |  | 1. Counseled on skin-to-skin contact/warmth | 1 | | | | | | 0 | | |
|  |  | 1. Checked baby for danger sign | 1 | | | | | | 0 | | |
|  |  | 1. Counseled on danger signs | 1 | | | | | | 0 | | |
|  |  | 1. Checked overt birth defects | 1 | | | | | | 0 | | |
|  |  | 1. Measured Temperature | 1 | | | | | | 0 | | |
|  |  | 1. Refereed to health center/hospital | 1 | | | | | | 0 | | |
|  |  | 99. Others | | | | | | | | | |
| 325 | Maternal satisfaction on the care by HEWs for her baby/her self | 1. Not satisfied 2. Satisfied 3. Do not want to speak | | | | | | | | | |
| 326 | Sometimes mothers after delivery have severe illnesses and should be taken immediately to a health facility.  What type symptoms would cause you to go to a health facility right away? **Do not read responses** | **M = mentioned, NM= not mentioned** | | **M** | | | | | | **NM** | |
|  |  | 1=Excessive vaginal bleeding | | 1 | | | | | | 0 | |
|  |  | 2=Foul-smelling discharge | | 1 | | | | | | 0 | |
|  |  | 3=High fever | | 1 | | | | | | 0 | |
|  |  | 4=Severe abdominal pain | | 1 | | | | | | 0 | |
|  |  | 5=Convulsions/coma | | 1 | | | | | | 0 | |
|  |  | 6=Excessive tiredness or breathlessness | | 1 | | | | | | 0 | |
|  |  | 7= sever head ache and/blurred vision | |  | | | | | | 0 | |
|  |  | 99=Others, specify: | | | | | | | | | |
| 327 | Sometimes newborns after delivery have severe illnesses and should be taken immediately to a health facility.  What type symptoms would cause your baby to go to a health facility right away? **Do not read responses** | **M = mentioned, NM= not mentioned** | | **M** | | | | | | **NM** | |
|  |  | 1=Convulsion of the newborn | | 1 | | | | | | 0 | |
|  |  | 2=Movement only when stimulated or no movement, even when stimulated/ unconscious | | 1 | | | | | | 0 | |
|  |  | 3=Not feeding well/poor suckling | | 1 | | | | | | 0 | |
|  |  | 4=Fast breathing (>=60 breaths per minute), | | 1 | | | | | | 0 | |
|  |  | 5=Grunting or severe chest in-drawing | | 1 | | | | | | 0 | |
|  |  | 6=Fever (>=37.5°C) newborn | | 1 | | | | | | 0 | |
|  |  | 7=Low body temperature (below 35.5°C) | | 1 | | | | | | 0 | |
|  |  | 8=Very small baby (less than 1500 grams or born more than two months early) | | 1 | | | | | | 0 | |
|  |  | 9=Any jaundice in first 24 hours of  life, or yellow palms and soles at any age | | 1 | | | | | | 0 | |
|  |  | 10=Cord bleeding/pus in the cord | | 1 | | | | | | 0 | |
|  |  | 11=Eyes red/swollen/discharge | | 1 | | | | | | 0 | |
|  |  | 12=Others, specify | | | | | | | | | |
| **Postnatal practices by the mother at home** | | | | | | | | | | | |
| 328 | Anything applied after cutting the cord? | 1. No 2. Yes | | If no, Q330 | | | | | | | |
| 329 | What was applied? | **M = mentioned, NM= not mentioned** | | **M** | | | | | | | **NM** |
|  |  | 1= Butter | | 1 | | | | | | | 0 |
|  |  | 2= Ash | | 1 | | | | | | | 0 |
|  |  | 3= chlorhexidane | | 1 | | | | | | | 0 |
|  |  | 4= cattle’s dung | | 1 | | | | | | | 0 |
|  |  | 5= oil | | 1 | | | | | | | 0 |
|  |  | 6= cold water | | 1 | | | | | | | 0 |
|  |  | 7=Others | | | | | | | | | |
| 330 | Time of baby bath | 1=Within 1 hour  2=1-23Hours  3=>24 hours  4 = not yet bathed | | | | | | | | | |
| 331 | In the first week of life, did you hold skin to skin against your breasts during the daytime and night time? | 1. No 2. Yes | | | | | | | | | |
| 332 | Did you ever breast feed your newborn? | 1. No 2. Yes | | | | If no, Q337 | | | | | |
| 333 | Time of initiation of breast feeding | 1. Within one hour 2. 1-24 hours 3. >24 hours 4. Not breast feed | | | | | | | | | |
| 334 | If Breast fed within one hour at what minutes started? | ___minute | | | | | | | | | |
| 335 | Frequency of breast feeding per day | Enter number | | | | | | | | | |
| 336 | Given colostrum to your newborn | 1. No 2. Yes | | | | | | | | | |
| 337 | Started complementary feeding (provided sugar, butter and/ juice) | 1. No 2. Yes | | | If no, Q339 | | | | | | |
| 338 | Age of child at first complementary food started | Enter in days | | |  | | | | | | |
| 339 | Vt K provided | 1. No 2. Yes | | |  | | | | | | |
| 340 | TTC eye ointment provided | 1. No 2. Yes | | |  | | | | | | |
|  | | | | | | | | | | | |
